# Molecular docking analysis of compounds from Andrographis paniculata with EGFR

**DOI:** 10.6026/97320630017023

**Published:** 2021-01-31

**Authors:** Anitha Roy

**Affiliations:** 1 Department of Pharmacology, Saveetha Dental College and Hospital, Saveetha Institute of Medical and Technical Sciences, Chennai, Tamil Nadu-600077, India

**Keywords:** Andrographis paniculata, EGFR, molecular docking studies

## Abstract

EGFR is linked with oral cancer. Therefore, it is of interest document the molecular docking analysis of compounds from Andrographis paniculata with EGFR. Data shows the binding features of five compounds 14- acetylandrographolide, andrograpanin, andrographolide, isoandrographolide and neoandrographolide from Andrographis paniculata with EGFR for further consideration.

## Background

Oral squamous cell carcinoma is the most predominant malignant epithelial neoplasm in the oral cavity [1]. Oral squamous cell carcinoma (OSCC) is caused by DNA mutations like other cancers; it is spontaneous but exacerbated by exposure to different forms of mutagens such as chemical, physical or microbial agents. Oral keratinocytes, the cell of origin of OSCC, may be transformed from a normal keratinocyte to a premalignant or potentially malignant keratinocyte by many DNA modifications, which would proliferate in a decreased restriction than normal, and grow cells independently to produce true cancer [2]. Oral squamous cell carcinomas arise from various anatomical locations of the oral cavity and oropharynx, but most commonly from the oral mobile tongue [3]. Approximately 50 % of oral cancers are caused by betel quid chewing in the increased betel quid-chewing region, 25 % by tobacco use (either smoking or chewing or both), 10-15 % by micronutrient deficiency and 7-19 % by alcohol consumption globally [4]. Nearly 95% of oral cancer is squamous cell carcinoma [5]. Global Cancer Data 2018 revealed that there have been approximately 354,864 new cases and 177,384 lip and oral cancer deaths in 2018; approximately 246,420 cases and 119,693 male and 108,444 cases and 57,691 female deaths have been recorded. OSCC incidence differs significantly across the regional region and more than half of all cancer instances happen in developing nations [6]. These troubling statistics on oral cancer illustrate the fact that, in an effort to reach a lower level of oral cancer worldwide, it is very important to establish an approach to OSCC care. In the majority of OSCC cases, epidermal growth factor receptor (EGFR) association (EGFR / ErB1 / HER1) has been reported to encourage aggressiveness, metastases, poor prognosis and resistance to anticancer therapy [7]. This is expressed in a variety of other cancers. EGFR is a tyrosine kinase receptor that belongs to the ErbB family and is an EGF receptor as well as an alpha-transforming growth factor. Resistance to chemotherapeutic agents used in the treatment of OSCC is linked with higher EGFR expression. They demonstrated resistance to drugs such as 5-fluorouracil, cisplatin, doxorubicin, and cyclophosphamide. Cetuximab is an authorised FDA drug commonly used to treat cancer by inhibiting EGFR [8]. Even though, such medications cannot be regarded as exceptional and are only successful as a first-line treatment choice in conjunction with platinum [9]. Andrographis paniculata is a herbaceous medicinal plant contributing to the Acanthaceae family and commonly known as the 'king of bitters' It is called as Nilavembu in the Tamil language. It has been commonly used for the treatment of flu, sore throat and upper respiratory tract infections in India and other Asian countries such as China, Thailand and Malaysia for decades. Therefore, it is of interest to document the molecular docking analysis of compounds from Andrographis paniculata with EGFR.

## Methodology

### Preparation of protein:

The target structure of EGFR with PDB ID: 2JIT was obtained from the Protein Data Bank. It belongs to the classification of Homo sapiens transferase proteins. The three-dimensional structure was determined using X-ray diffraction process. The recovered protein structure was prepared using AutoDock Methods. Water molecules and all non-standard residues have been excluded from the initial structure. Then, all missing hydrogens and kollman charges have been applied to the device; the prepared protein receptor was then saved as a pdbqt format and stored directly in the PyRx workspace directories.

### Ligand Preparation:

Ten chemical components of Andrographis paniculata have been obtained from the Pubchem database (Table 1 - see PDF). In this analysis, pdb coordinates were used for all hydrogen output formats. The charges were further repaired by inserting partial gasteiger charges and then push the autodock. Then the structure of the compounds was opened on PyRx by clicking on Load Molecule and making ligand.

### Molecular docking studies:

Many docking algorithms becomes capable of constructing a wide range of possible structures, so they still need a means to score each structure to categories those of greatest interest. In the present study, the docking process was carried using PyRx 0.8 with the Autodock Vina method using the Lamrkian genetic algorithm as the score function was completed [10]. Possession of the ligands located on the basis of the highest binding energy. The PyMol molecular viewer (http:/www.pymol.org/) was used for the study of docked structures.

## Results and Discussion:

In this analysis, the ligand-protein molecular docking simulation was used for preliminary investigation and confirmation of potential compounds for the selected EGFR target. The analysis of the effective docked ligands against the selected target demonstrated the binding mode of the compounds involved in this research and verified their function as anti-cancer agents. The binding energy of the drug – protein (receptor) interactions is important to explain how well the drug binds to the target macromolecule. The residues that participated in the formation of hydrogen bonds inside the active binding site region highlighted the importance of these residues to the reported binding energy with respect to the hit found against the EGFR target protein. The resulting hypothesis may be an incredible starting point for the creation of some new pathways as potential EGFR inhibitors that improve affinity as well as intrinsic function. The results of this work show that powerful analytical tools are capable of recognizing potential ligands. The use of computational methods in the discovery and creation of drugs could be used to minimize time and minimize the work of a medical chemist. The molecular docking simulation of the phytochemicals provided by Andrographis paniculata reveals that perhaps the plant constituents all have comparatively high binding energy and therefore low binding (Table 2 - see PDF). Out of ten compounds, best five compounds (14-acetylandrographolide, Andrograpanin, Andrographolide, Isoandrographolide & Neoandrographolide) were selected based on scoring parameters.

**Figure 1:**
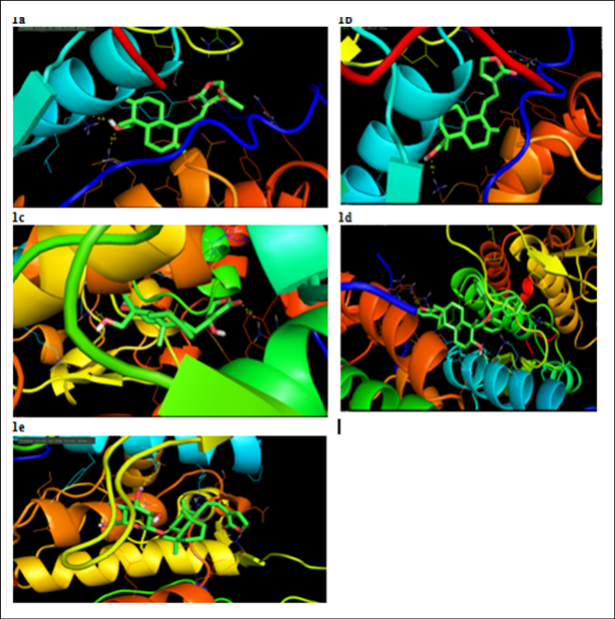
Molecular interaction of EGFR with a) 14-acetylandrographolide b) Andrograpanin c) Andrographolide d) Isoandrographolide e) Neoandrographolide.

The 14-acetylandrographolide bound to the target exhibited a fitness score of -8.0 and interacted with the active site residues ASN-700, ASP-761, TYR-764 & LYS-949. The compound andrographolide exhibited a fitness score of -7.1 and interacted with the residues LYS-949 & ARG-977 at the active site. This shows good interaction and efficient score (Table 2 - see PDF). The compounds Isoandrographolide showed the good binding affinity with the binding score of -8.1 and formed the two hydrogen bond interactions with EGFR protein through ASP-761 & ARG-977. Likewise Neoandrographolide and Andrograpanin showed the strongest binding score -7.0 & 6.8 respectively. In this Neoandrographolide formed three hydrogen bonds with the amino acids ASP-761, LYS-949 & LYS-960 and Andrograpanin showed the two hydrogen bonds with EGFR through LYS-949 & ARG-977. Selected five docked complexes showed the hydrogen bonds distance below 3 Å it confirmed that all the compounds formed the stable complex with EGFR. Analysis of these complexes also revealed that all most all the compounds formed the hydrogen with the amino acids LYS-949 & ARG-977 (Figure 1). So, these amino acids might be responsible for functional of the target protein. Further experimentally analysis is needed to confirm this finding.

## Conclusion

Data shows the binding features of five compounds 14-acetylandrographolide, andrograpanin, andrographolide, isoandrographolide and neoandrographolide from Andrographis paniculata with EGFR for further consideration.
